# Electromechanical Conditioning of Adult Progenitor Cells Improves Recovery of Cardiac Function After Myocardial Infarction

**DOI:** 10.5966/sctm.2016-0079

**Published:** 2016-09-29

**Authors:** Aida Llucià‐Valldeperas, Carolina Soler‐Botija, Carolina Gálvez‐Montón, Santiago Roura, Cristina Prat‐Vidal, Isaac Perea‐Gil, Benjamin Sanchez, Ramon Bragos, Gordana Vunjak‐Novakovic, Antoni Bayes‐Genis

**Affiliations:** ^1^ Heart Failure and Cardiac Regeneration Research Programme, Health Science Research Institute Germans Trias i Pujol, Badalona, Spain; ^2^ Center of Regenerative Medicine in Barcelona, Barcelona, Spain; ^3^ Electronic and Biomedical Instrumentation Group, Departament d’Enginyeria Electrònica, Universitat Politècnica de Catalunya, Barcelona, Spain; ^4^ Department of Neurology, Division of Neuromuscular Diseases, Beth Israel Deaconess Medical Center, Harvard Medical School, Boston, Massachusetts, USA; ^5^ Department of Biomedical Engineering, Columbia University, New York, New York, USA; ^6^ Department of Medicine, Columbia University, New York, New York, USA; ^7^ Cardiology Service, Hospital Universitari Germans Trias i Pujol, Badalona, Spain; ^8^ Department of Medicine, Universitat Autònoma de Barcelona, Bellaterra, Spain

**Keywords:** Biophysical stimulation, Cardiac adipose tissue‐derived progenitor cells, Tissue engineering, Cardiac regeneration, Electromechanical conditioning, Myocardial infarction

## Abstract

Cardiac cells are subjected to mechanical and electrical forces, which regulate gene expression and cellular function. Therefore, in vitro electromechanical stimuli could benefit further integration of therapeutic cells into the myocardium. Our goals were (a) to study the viability of a tissue‐engineered construct with cardiac adipose tissue‐derived progenitor cells (cardiac ATDPCs) and (b) to examine the effect of electromechanically stimulated cardiac ATDPCs within a myocardial infarction (MI) model in mice for the first time. Cardiac ATDPCs were electromechanically stimulated at 2‐millisecond pulses of 50 mV/cm at 1 Hz and 10% stretching during 7 days. The cells were harvested, labeled, embedded in a fibrin hydrogel, and implanted over the infarcted area of the murine heart. A total of 39 animals were randomly distributed and sacrificed at 21 days: groups of grafts without cells and with stimulated or nonstimulated cells. Echocardiography and gene and protein analyses were also carried out. Physiologically stimulated ATDPCs showed increased expression of cardiac transcription factors, structural genes, and calcium handling genes. At 21 days after implantation, cardiac function (measured as left ventricle ejection fraction between presacrifice and post‐MI) increased up to 12% in stimulated grafts relative to nontreated animals. Vascularization and integration with the host blood supply of grafts with stimulated cells resulted in increased vessel density in the infarct border region. Trained cells within the implanted fibrin patch expressed main cardiac markers and migrated into the underlying ischemic myocardium. To conclude, synchronous electromechanical cell conditioning before delivery may be a preferred alternative when considering strategies for heart repair after myocardial infarction. Stem Cells Translational Medicine *2017;6:970–981*

**Significance Statement**

Cardiac cells are constantly subjected to mechanical and electrical signals. Hence, in vitro electromechanical stimuli could benefit further cell integration and retention into the myocardium. Electromechanical conditioning of adipose‐derived progenitor cells promoted a cardiogenic‐like phenotype in vitro, which drove cardiac function recovery and increased vessel density when tested in a myocardial infarction model in vivo. This is the first study to report the benefits of electromechanically stimulated cells in an in vivo scenario. This physiological strategy seems promising to recover cardiac function after myocardial infarction and is ready for preclinical testing and clinical use.

## Introduction

Cardiovascular diseases remain the most common cause of mortality worldwide [1]. Myocardial infarction (MI) leads to irreversible sequelae of events leading to impaired heart function and, in some cases, to shorter lifespan [2]. Heart transplantation, a valid therapeutic option for patients with a terminally failing heart, is hampered by the low number of donor organs [3], whereas mechanical ventricular assist devices are mostly used as a bridge to transplant [4]. With the first clinical trial, cardiac tissue engineering is now emerging as a new therapeutic modality [5]. Most cardiac tissue‐engineering approaches combine cells with biomaterials in a three‐dimensional (3D) context [6].

In the developing and adult heart, cardiac cells are constantly subjected to cyclic loading induced by electrical signals. Mechanical stretch improves contractility [7], facilitates secretion of growth factors and calcium handling in cardiomyocytes, and modifies extracellular matrix synthesis in cardiac fibroblasts [8]. In general, mechanical stretch seems to improve heart muscle survival, cell alignment, elongation, hypertrophy, and differentiation [9]. The associated electrical signals are crucial for synchronized contraction of cardiac muscle, enhancing impulse propagation, ultrastructural organization, cell elongation, and alignment and the formation of functional gap junctions [9, 10, 11, 12, 13]. Accordingly, synchronous application of electrical and mechanical stimuli has the potential to benefit engineered cardiac construct properties. Nevertheless, electromechanical conditioning remains a relatively unexploited technology, and the efforts are more focused on engineered heart tissues suitable for disease modeling than on cell therapy purposes [14, 15]. We recently optimized an electromechanical stimulation protocol for engineering heart muscle constructs from neonatal rat heart cells, achieving a positive force‐frequency relationship [15].

The optimal cell lineage for cardiac regeneration is yet to be defined. Our group has characterized a population of human adipose tissue‐derived progenitor cells of cardiac origin (cardiac ATDPCs). Cardiac ATDPCs reside in the epicardial fat and display cardiac and endothelial differentiation potential, as well as beneficial histopathological and functional effects, both when injected as a cell solution into the infarct border zone [16] and when introduced within a fibrin patch [17]. Here we investigated electromechanical conditioning of cardiac ATDPCs before encapsulation in a fibrin scaffold that was implanted onto ischemic myocardium of infarcted mice to boost cardiac function.

## Materials and Methods

### Human ATDPC Isolation and Culture

Human ATDPCs were isolated from cardiac (cardiac ATDPCs) and subcutaneous (subcutaneous ATDPCs) adipose tissues obtained from patients undergoing cardiac surgery, under a protocol approved by the Germans Trias i Pujol University Hospital Ethics Committee. Informed consent was obtained from all patients, and the study protocol conformed to the principles of the Declaration of Helsinki. Tissues were obtained from a total of 11 patients (cardiac adipose tissue) and 6 patients (subcutaneous adipose tissue). The cells isolated from each tissue source were pooled and used for experiments. Subcutaneous ATDPCs were used as control cells for cardiac ATDPC in vitro experimentation.

Adipose tissue biopsy samples were harvested and processed as described previously [16]. Briefly, samples were rinsed with phosphate‐buffered saline (PBS) and cut into small pieces, and visible blood vessels were removed; next, cells were isolated by collagenase II (Thermo Fisher Scientific Life Sciences, Waltham, MA, http://www.thermofisher.com) digestion. Adhered cells were grown in α‐minimum essential medium (Sigma‐Aldrich, St. Louis, MO, http://www.sigmaaldrich.com) supplemented with 10% fetal bovine serum (Thermo Fisher Scientific Life Sciences), 1 mM l‐glutamine (Thermo Fisher Scientific Life Sciences), and 1% penicillin/streptomycin (Thermo Fisher Scientific Life Sciences), and cultured under standard conditions (37°C and 5% CO_2_).

### Electromechanical Stimulation Device

**Figure 1:**
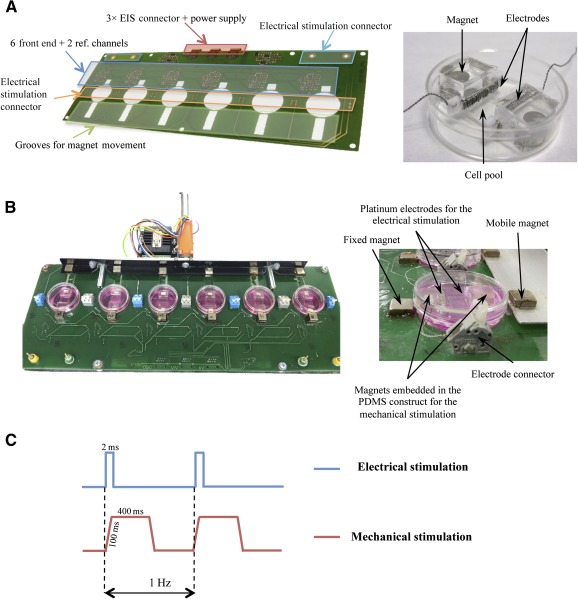
Electromechanical stimulator. **(A):** SolidWorks design of the printed circuit board used to perform electromechanical conditioning on six 3.5‐cm culture plates (left), and detail of a PDMS silicone substrate (right). **(B):** Electromechanical stimulator (left) and side view magnification (right). **(C):** Diagram showing the stimulation regimen applied to the cultured cells: electrical (blue) and mechanical (red) stimulations. Pulse durations and periods, delay between electrical and mechanical pulses, and mechanical pulse shape were programmable through a LabView custom application. Abbreviations: EIS, electrical impedance spectroscopy; PDMS, polydimethylsiloxane; ref., reference.

The custom‐made electromechanical stimulation unit is an improvement of the electrical stimulation setup already described [10]. This one consisted of a combination of a monophasic programmable electrical device, a printed circuit board that facilitated the robust connection of the electrodes, and a biocompatible polydimethylsiloxane (PDMS) (Sylgard 184, Dow Corning Corp., Washington, DC, https://www.dowcorning.com) silicone construct designed to provide structural support to cells, electrodes and magnets (Fig. 1A, 1B). The disposable stimulation silicone accommodates a cell culture pool in a flexible area (1 cm × 1 cm × 2 mm) and holds two electrodes built with platinum wire wrapped around a polytetrafluoroethylene core, placed at two opposing sides of the flexible area creating an electric field to induce electrical stimulation. To provide a mechanical stimulation simultaneously and synchronously with the electrical one, two neodymium magnets (Supermagnete, Gottmadingen, Germany, https://www.supermagnete.de/eng) were embedded in the PDMS constructs (Fig. 1A, 1B). The use of magnets enables the performance of noninvasive mechanical stimulation. Two additional magnets are placed outside the culture plates. One of them is static and fixes one end of the silicone construct, and the second one pulls the other end, following a programmable pattern from a computer‐controlled linear motor (LM 2070‐040‐11 + MCLM3006S, Faulhaber, Schönaich, Germany, https://www.faulhaber.com). The device has six parallel channels to provide stimulation to up to six culture plates and permits the combination of both electrical and mechanical stimulation, either independently or synchronously. Pulse amplitudes, durations and periods, delay between electrical and mechanical pulses, and mechanical pulse shape are programmable through a LabView (National Instruments, Austin, TX, http://www.ni.com) custom application (Fig. 1C).

### Cell Culture With Electromechanical Conditioning

Concisely, 3 × 10^4^ cells were seeded on each PDMS construct 1 day before the beginning of the stimulation. The conditioning lasted 7 days, and unstimulated cells were used as a control for electromechanical conditioning, while subcutaneous ATDPCs were used as a control for cardiac ATDPCs. The electromechanical conditioning protocol consisted of alternating current 2‐millisecond monophasic square‐wave pulses of 50 mV/cm at 1 Hz and 10% stretching for 7 days. This was conducted six times (with at least three replicates each) for the in vitro experimentation (gene and protein analyses), and 7 days was the endpoint. Immunostainings were performed on the cells attached to the PDMS construct and fixed with 10% formalin at day 7. Gene analyses were carried out after the trypsinization of the cells attached to the PDMS construct for both control and stimulated groups (*n* = 6).

For the animal studies, cardiac ATDPCs were harvested from stimulated or unstimulated PDMS constructs after 7 days, and the fibrin patch was immediately produced and kept under standard culture conditions for less than 24 hours before it was implanted. Briefly, 1 × 10^5^ cells were mixed with 8 μl of fibrinogen solution (70–110 mg/mL), followed by the addition of 8 μl of thrombin solution (500 UI/mL) for jellification (Tissucol duo; Baxter, Utrecht, The Netherlands, http://www.baxter.nl). The area of the fibrin patch was ∼7 mm^2^ and ∼1 mm in height. Fibrin patches were freshly produced in nine sequential procedures (one procedure every surgery day) for the whole in vivo experimentation (*n* = 39 animals), and 21 days was the endpoint. Echocardiographic measurements were acquired at baseline (2 days before the surgery), post‐MI (2 days after the surgery), and at presacrifice (21 days after the surgery) for all animals.

### Animal Studies

The animal study protocol was approved by the Institutional Animal Care and Use Committee (CSIC‐ICCC, Cardiovascular Research Center) and complied with guidelines concerning the use of animals in research and teaching, as defined by the Guide for the Care and Use of Laboratory Animals (NIH Publication No. 80‐23). All procedures were performed in accordance with both the national and European legislation (Spanish Royal Decree RD 53/2013 and EU Directive 2010/63/EU) for the protection of animals used for research experimentation.

### MI Model and Fibrin‐Cell Patch Delivery

Briefly, mice were anesthetized with a mixture of O_2_/isoflurane (2%) (Baxter), intubated, and mechanically ventilated (90 breaths per minute, 0.1‐ml tidal volume) by using a SAR830/AP small animal ventilator (CWE, Inc., Ardmore, PA, http://www.cwe‐inc.com). An anterior thoracotomy was performed, and the proximal left anterior descending (LAD) coronary artery was occluded by using a 7‐0 silk suture. Sham animals were operated on in the same manner with no occlusion of the LAD coronary artery before implantation of the fibrin‐cell patches. To generate the adhesive construct, Tissucol solution (8 μl) with 1 × 10^5^ cells or culture medium was mixed with 8 μl of thrombin solution for jellification (Tissucol duo; Baxter). Fibrin patches with or without cardiac ATDPCs were implanted by using Glubran surgical glue (Cardiolink, Barcelona, Spain, http://www.cardiolink.es), which fulfills the required safety and compatibility standards for use in experimental animals and humans, to seal the edge of the patch to the myocardium. The animals were sacrificed 21 days after the operation. By using cardioplegic solution, hearts were arrested in diastole and then excised, fixed in 10% formalin solution (Sigma‐Aldrich), cryopreserved in 30% sucrose in PBS, embedded in optimum cutting temperature (Sakura Finetek Europe B.V., AV Alphen aan Den Rijn, The Netherlands, http://www.sakura.eu), and snap‐frozen in liquid nitrogen‐cooled isopentane for histological analysis.

### Experimental Groups

The study was performed on 39 female SCID mice (11–15 weeks old and weighing 20–25 g; Charles River Laboratories, Frederick, MD, http://www.criver.com) using cardiac ATDPCs. Cells were labeled before fibrin patch inclusion by using the PKH26 Red Fluorescent Cell Linker Kit for General Cell Membrane Labeling (Sigma‐Aldrich) following manufacturer’s protocol. Mice were distributed randomly into the following groups: MI alone (MI) (*n* = 8), MI with cell‐free fibrin implants (MI+Fibrin) (*n* = 6), MI with implantation of fibrin loaded with naïve control cardiac ATDPCs (MI+Con) (*n* = 8), and MI with implantation of fibrin loaded with electromechanically conditioned (EMC) cardiac ATDPCs (MI+EMC) (*n* = 5). Sham groups that lacked MI and underwent implantation of control fibrin‐cell patches (Sham+Con) (*n* = 7) and EMC fibrin‐cell patches (Sham+EMC) (*n* = 5) served as control groups to obtain the effects of cardiac ATDPCs on healthy myocardium. The global mortality in the experiment was only 7.14% (three mice: one MI, one MI+Con, and one Sham+Con).

### Quantitative Real‐Time Reverse‐Transcriptase Polymerase Chain Reaction

Total RNA was isolated from cardiac and subcutaneous ATDPCs by using the AllPrep RNA/Protein Kit (Qiagen, Hilden, Germany, http://www.qiagen.com). cDNA was synthesized by using random hexamers (Qiagen) and the iScript One‐Step reverse‐transcriptase polymerase chain reaction (RT‐PCR) kit (Bio‐Rad, Hercules, CA, http://www.bio‐rad.com) according to the manufacturer's protocol. cDNA was preamplified with the TaqMan PreAmp Master Mix Kit (Applied Biosystems, Foster City, CA, http://www.appliedbiosystems.com) and then diluted 1:5 with RNase‐free water.

Real‐time PCR amplifications were performed with 2.5 µl of cDNA in a final volume of 10 µl, containing 5 µl of TaqMan 2× Universal PCR Master Mix, 2 µl of RNase‐free water, and 0.5 µl of 6‐carboxyfluorescein amidite (6‐FAM)‐labeled primer/probe (Applied Biosystems), including glyceraldehyde‐3‐phosphate dehydrogenase (GAPDH) (Hs99999905_m1), T‐box transcription factor (Tbx5) (Hs00361155_m1), myocyte‐specific enhancer factor 2A (MEF2A) (Hs01050409_m1), GATA‐binding protein 4 (GATA‐4) (Hs00171403_m1), α‐actinin (*ACTN1* gene) (Hs00241650_m1), cardiac troponin I (cTnI, *TNNI3* gene) (Hs00165957_m1), connexin43 (Cx43, *GJA1* gene) (Hs00748445_s1), sarco/endoplasmic reticulum Ca^2+^‐ATPase (SERCA2, *ATP2A2* gene) (Hs00544877_m1), and β‐myosin heavy chain 7 (β‐MyHC, *MYH7* gene) (Hs00165276_m1). The following cardiac markers were evaluated: transcription factors (Tbx5, MEF2A, and GATA‐4), structural markers (α‐actinin, cTnI, and β‐MyHC), and calcium handling‐related markers (Cx43 and SERCA2).

Data were collected and analyzed in duplicate on the Light Cycler 480 Real‐Time PCR System (Roche, Indianapolis, IN, http://www.roche.com). The Livak method was used to quantify the absolute (2^−ΔΔCT^) and relative (2^−ΔCT^) expression of each gene between electromechanically conditioned and control samples, using GAPDH as an endogenous reference.

### Immunofluorescence and Morphometric Examination

Cells attached to the PDMS construct were fixed with 10% formalin, permeabilized, blocked in 10% normal horse serum for 1 hour, and incubated for 1 hour at room temperature with primary antibodies raised against Cx43 (6.4 µg/ml; Sigma‐Aldrich), sarcomeric α‐actinin (11.5 µg/ml ascites fluid; Sigma‐Aldrich), GATA‐4 (4 µg/m.; R&D Systems Inc., Minneapolis, MN, https://www.rndsystems.com), MEF2 (4 µg/ml; Santa Cruz Biotechnology, Santa Cruz, CA, http://www.scbt.com), and SERCA2 (4 µg/ml; Santa Cruz Biotechnology). Secondary antibodies were conjugated with Cy2 and Cy3 (7.5 µg/ml; Jackson ImmunoResearch, West Grove, PA, https://www.jacksonimmuno.com), and actin fibers (actinF) were stained with Phalloidin Alexa 568 (0.161 µM; Thermo Fisher Scientific Life Sciences). Nuclei were counterstained with 4′,6‐diamidino‐2‐phenylindole (DAPI) (0.1 µg/ml; Sigma‐Aldrich). Images were acquired with the Axio Observer Z1 inverted microscope (Zeiss, Stuttgart, Germany, http://www.zeiss.com).

Hearts were cross‐sectioned from apex to base (10‐μm‐thick sections spaced every 300 μm). Eight serial cryosections per animal were stained with Masson’s trichrome (collagen, green; myocardium, red; nuclei, black or brown; cytoplasm, pink) for morphometry. All sections were blindly examined and photographed using a SMZ 800 stereoscope (Nikon, Tokyo, Japan, http://www.nikon.com). Infarct size volume, expressed as a percentage of the total left ventricle (LV) wall volume, was calculated by addition of partial scar volumes of each section. Scar thickness was calculated as the mean of at least three measurements made in three different sections for each animal. Additionally, Gallego’s modified trichromic (collagen, blue; myocardium, yellow‐pink; elastic fibers, purple; nuclei, fuchsia) and Movat’s pentachromic (nuclei, black; collagen, yellow; ground substance, blue; muscle, purple; elastic fibers, brownish gray) stainings were carried out on heart cross‐sections from all groups.

Further immunoanalyses were performed on cryosections by using specific monoclonal antibodies against Cx43 (Sigma‐Aldrich), sarcomeric α‐actinin (Sigma‐Aldrich), GATA‐4 (R&D Systems), MEF2 (Santa Cruz Biotechnology), SERCA2 (Santa Cruz Biotechnology), biotinylated GSLI B4 isolectin (10 µg/ml; Vector Laboratories, Burlingame, CA, http://vectorlabs.com), cTnI (10 µg/ml; Abcam, Cambridge, MA, http://www.abcam.com), phospho‐histone H3 (PH3) (1 µg/ml; Cell Signaling Technology, Beverly, MA, http://www.cellsignal.com), CD31 (4 µg/ml; Abcam), smooth muscle actin (SMA) (1:50 ascites fluid; Sigma‐Aldrich), and vimentin (10 µg/ml; Abcam). Secondary antibodies were conjugated with Cy2 and Cy3 (Jackson ImmunoResearch). Streptavidin was conjugated with Alexa 488 (Thermo Fisher Scientific Life Sciences). Nuclei were counterstained with DAPI (Sigma‐Aldrich). Images were captured under a laser confocal microscope (Axio‐Observer Z1; Zeiss). Quantitative histological measurements were made by using ImageJ analysis software (NIH, Bethesda, MD, https://imagej.nih.gov).

### Cell Viability Analysis

To determine cell viability in the fibrin patches, the Live/Dead viability/cytotoxicity kit (Thermo Fisher Scientific Life Sciences) was used according to the manufacturer’s instructions. Fibrin patches loaded with 1 × 10^5^ cells were cultured for 3 weeks under standard culture conditions and then washed in PBS before staining. The stained patch constructs were analyzed and quantified by using the confocal microscope (Axio‐Observer Z1; Zeiss), and Maximum Projection Intensity plus Tiles‐stitching image postprocessing were applied (Zen Blue software; Zeiss).

### Vessel Density

The vessel area was assessed in sections stained with biotinylated GSLI B4 isolectin (10 µg/ml; Vector Laboratories) for border and remote regions from the scar in all animals, in which at least six fields per region were counted by an investigator blinded to the treatment groups. For sham animals, because the scar is absent, the border region corresponds to the patch margin, and the remote region to the opposite and furthest region from the patch. Lectin staining was measured with the ImageJ analysis software (NIH) and normalized by its field area. Next, the border value was corrected by the remote one, and this was represented as the vessel density percentage.

### Analysis of Cardiac Function

Cardiac function was assessed by echocardiography using an 18‐ to 38‐MHz linear‐array transducer with a digital ultrasound system (Vevo 2100 Imaging System, VisualSonics, Toronto, Ontario, Canada, http://www.visualsonics.com). Measurements were made at baseline, 2 days after MI, and 21 days after surgery (presacrifice) for all 39 animals. The investigators were blinded to the treatment groups. Standard parasternal short‐axis views were obtained in B and M modes. Functional parameters were measured, including left ventricle fractional shortening, LV ejection fraction (LVEF), LV anterior wall thickness, LV posterior wall thickness, LV end‐diastolic dimension, and LV end‐systolic dimension.

### Statistical Analysis

Relative fold changes of cardiac and subcutaneous ATDPC gene expressions were compared by using Student’s *t* test, and the statistical difference was determined for the samples from six separate experiments. Vessel density and morphometry were assessed by using one‐way analysis of variance (ANOVA) and Tukey post hoc analysis for multiple comparisons. Greenhouse‐Geisser analysis was used for LVEF repeated measures (baseline and post‐MI) to confirm homogeneity of surgical procedure. Paired‐samples *t* test was also used to compare differences between baseline and presacrifice echocardiographic parameters in each experimental group. The LVEF differentials (ΔLVEF) between presacrifice and baseline were evaluated by using one‐way ANOVA and Tukey post hoc analysis for multiple comparisons. All the results are presented as the mean ± SEM; ∗, *p* < .05 was considered statistically significant. Statistical analyses were performed by using SPSS Statistics software (version 21, IBM, Armonk, NY, http://www.ibm.com).

## Results

### Electromechanical Conditioning

Electrical and mechanical conditionings were first applied individually to optimize each protocol. Electrical conditioning was based on a previous publication in which alternating current 2‐millisecond monophasic square‐wave pulses of 50 mV/cm at 1 Hz were found optimal [10]. Synchronous mechanical conditioning was performed with an ad hoc custom magnet‐driven system (Materials and Methods) operated at a frequency of 1 Hz and a strain of 10% for 7 days (Fig. 1; supplemental online Movie 1). Briefly, 3 × 10^4^ cells were seeded on the silicone surface and subjected to stimulation, with changes of culture medium twice a week. Unstimulated cells were used as a control for electromechanical conditioning. Subcutaneous ATDPCs were used as a control for cardiac ATDPCs.

### Physiological Conditioning Promotes Expression of Cardiac Genes and Proteins

**Figure 2:**
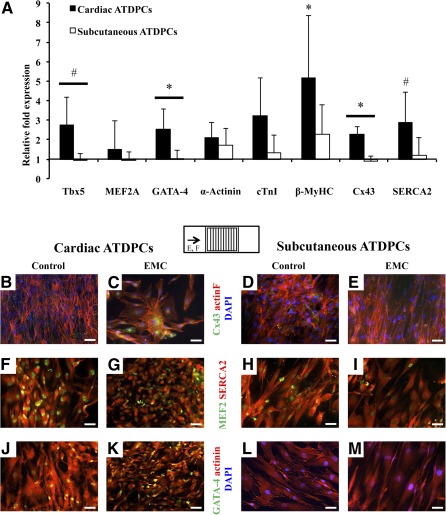
Gene and protein analyses after electromechanical conditioning of ATDPCs. 3 × 10^4^ cells were seeded on each polydimethylsiloxane construct 1 day before the beginning of the stimulation, and the cells attached were used for gene and protein analyses. **(A):** Real‐time polymerase chain reaction of main cardiac genes in cardiac and subcutaneous ATDPCs. Relative expression of cardiomyogenic markers in EMC versus nonconditioned controls is shown for cardiac and subcutaneous ATDPCs. Values were normalized to glyceraldehyde‐3‐phosphate dehydrogenase expression and are shown as mean ± SEM for six independent experiments. #, *p* < .1 (trend); ∗, *p* < .05 (significance) versus the subcutaneous ATDPC group. **(B–M):** Protein expression in cardiac and subcutaneous ATDPCs on a vertical patterned surface, perpendicular to the electric field (E) and stretching (force; F), as shown on the superior drawing. Phalloidin staining (actin F; red) and Cx43 expression (green), SERCA2 (red), MEF2 (green), sarcomeric α‐actinin (red), and GATA‐4 (green) expression in control **(B, D, F, H, J, L)** and EMC **(C, E, G, I, K, M)** cardiac (left) and subcutaneous (right) ATDPCs. Nuclei were counterstained with DAPI (blue; **B–E, L, M**). Scale bars = 50 µm. Abbreviations: β‐MyHC, β‐myosin heavy chain 7; ATDPCs, adipose tissue‐derived progenitor cells; cTnI, cardiac Troponin I; Cx43, connexin43; DAPI, 4′,6‐diamidino‐2‐phenylindole; EMC, electromechanically conditioned; GATA‐4, GATA‐binding protein 4; MEF2A, myocyte‐specific enhancer factor 2A; SERCA2, sarco/endoplasmic reticulum Ca^2+^‐ATPase; Tbx5, T‐box transcription factor.

Electromechanical conditioning modulated gene expression in both cell types, with cardiac ATDPCs showing a stronger upregulation of cardiac genes (Fig. 2A). In cardiac ATDPCs, Tbx5 (*p* = .081), GATA‐4 (*p* = .050), and Cx43 (*p* = .025) increased approximately twofold compared with subcutaneous ATDPCs, in response to electromechanical conditioning. Additionally, significant and higher increases were observed on key structural and calcium‐related genes, such as β‐MyHC (fivefold, *p* = .000) and SERCA2 (2.8‐fold, *p* = .076) in cardiac ATDPCs EMC samples compared with controls.

The protein expression of main cardiac markers in nonconditioned controls and EMC ATDPCs is shown in Figure 2B–2M. Cx43 was mostly distributed in the cytoplasm and at the plasma membrane to allow cell connections through gap junctions (Fig. 2B–2E). MEF2 was expressed in the nuclei, and SERCA2 and α‐actinin were observed in the cytoplasm without mature sarcomere organization or synchronous beating (Fig. 2F–2M). GATA‐4 protein expression was detected only in the nuclei of control and EMC cardiac ATDPCs (Fig. 2J, 2K).

### Viability and Migration of Physiologically Trained Cardiac ATDPCs in 3D Fibrin Gel

**Figure 3:**
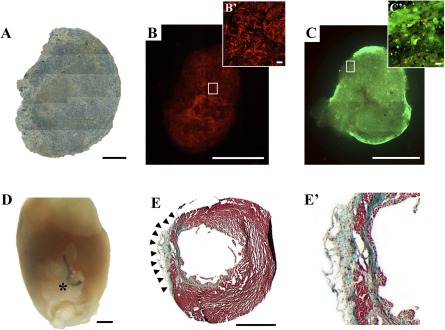
Macroscopic evaluation of the three‐dimensional engineered construct with cardiac adipose tissue‐derived progenitor cells (cardiac ATDPCs) cultured for 21 days before in vivo grafting. **(A):** Brightfield image composition of the cellular fibrin patch under standard culture conditions. **(B, B′):** PKH26 cell labeling (red) of cardiac ATDPCs in the fibrin patch **(B)** and magnification of the core region **(B′)**. **(C):** Representative image showing cell viability (green cells, alive; red cells, dead) in a fibrin patch loaded with cardiac ATDPCs, as performed by the live/dead assay. **(C′):** Magnification of a construct border zone with an abundance of viable cells (green). **(D):** Representative photograph of an excised heart from a postinfarction animal (visible ligation) 21 days after the cellular fibrin construct implantation (asterisk). **(E, E′):** Representative image of Masson’s trichromic staining of heart cross‐section from a myocardial infarction plus electromechanically conditioned‐treated animal **(E)** and its magnification **(E′)**. Scale bars = 1 mm **(A–E)** and 20 µm **(B′, C′)**.

Cardiac ATDPCs were labeled with PKH26 and cultured in vitro in a 3D fibrin gel to assess cell tracking and viability before in vivo implantation. After 21 days of in vitro culture, the fibrin patch maintained its initial morphology and size (Fig. 3A), and the cells retained the PKH26 labeling (Fig. 3B). Importantly, ∼84% of cells (green fluorescence) remained viable (Fig. 3C).

The engineered fibrin construct was implanted over the infarcted area in the murine model of acute MI (Fig. 3D) and over healthy myocardium (sham animals). After 21 days, the physiologically engineered construct (MI+EMC) was nicely attached to the myocardium and almost indiscernible through macroscopic observation (Fig. 3D, 3E). Construct’s adaptation to the murine myocardium surface is shown in different heart cross‐sections of MI+EMC animals (Fig. 3E; supplemental online Fig. 1).

**Figure 4:**
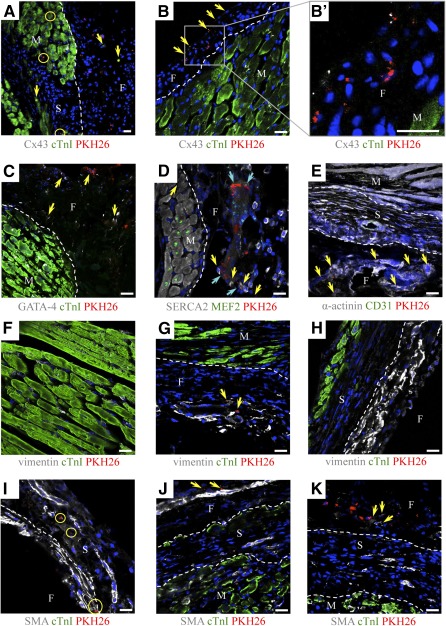
Migration and differentiation of electromechanically conditioned cardiac adipose tissue‐derived progenitor cells (cardiac ATDPCs) in a mouse model of myocardial infarction. Immunofluorescence analysis of heart cross‐sections at 21 days for cTnI (green) and Cx43 (gray) **(A–C)**; GATA‐4 (gray) and cTnI (green) **(C)**; SERCA2 (gray) and MEF2 (green) **(D)**; sarcomeric α‐actinin (gray) and CD31 (green) **(E)**; vimentin (gray) and cTnI (green) **(F–H)**; and SMA (gray) and cTnI (green) **(I–K)**. Cardiac ATDPCs were labeled with PKH26 (red fluorescence), and nuclei were counterstained with 4′,6‐diamidino‐2‐phenylindole (blue). Circles indicate the migration of cardiac ATDPCs into murine tissue. Arrowheads show representative expressions of the protein of interest in each panel. Scale bars = 20 µm. Abbreviations: cTnI, cardiac Troponin I; Cx43, connexin43; F, fibrin; GATA‐4, GATA‐binding protein 4; M, myocardium; MEF2A, myocyte‐specific enhancer factor 2A; S, scar; SERCA2, sarco/endoplasmic reticulum Ca^2+^‐ATPase; SMA, smooth muscle actin.

Cardiac ATDPCs migration to underlying ischemic myocardium was observed only occasionally in both control and EMC fibrin constructs (Fig. 4A, 4I). De novo expression of cTnI was identified in some of the implanted cells (Fig. 4A). Cardiac ATDPCs within the fibrin patch also contained Cx43, GATA‐4, SERCA2, MEF2, and α‐actinin (Fig. 4A–4E), suggesting that the cardiomyogenic lineage gained in vitro persisted in vivo. Additional proof of cell phenotype is the absence of PH3, associated with proliferation, from cardiac ATDPCs (data not shown). Moreover, DAPI staining showed adequate morphology of cell nuclei, evidencing that cells remained alive.

Remarkably, murine cell migration between the host tissue and the engineered construct was observed in all treated groups and was most obvious in animals in which a cell‐free fibrin hydrogel was implanted (supplemental online Fig. 2). Analysis of the colonized spindle‐shaped cell population observed in the fibrin patch was positive for vimentin and smooth muscle actin, suggesting the presence of fibroblasts and myofibroblasts (Fig. 4F–4K; supplemental online Fig. 2).

### Neovascularization of Engineered Fibrin Constructs and Underlying Myocardium

**Figure 5:**
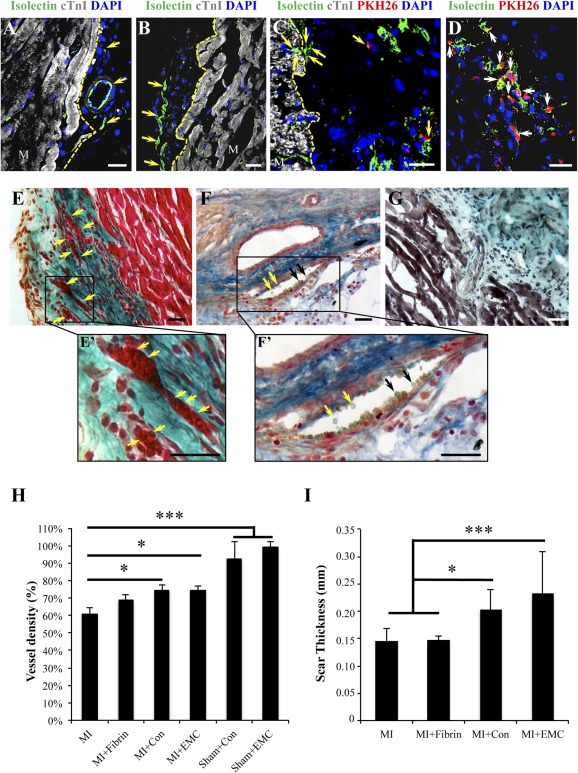
Vascular analysis**. (A–D):** GSLI B4 isolectin staining (green) showing vessel‐like structures within the fibrin construct at 21 days after infarction, as well as in the myocardium‐fibrin interphase **(A, B)**. An isolectin‐positive vessel (green) connects the murine myocardium stained with cTnI (gray) and the fibrin patch **(C)**. Cardiac ATDPCs (red) colocalization with vascular structures GSLI B4 isolectin positive (green) **(C, D)**. Nuclei were counterstained with DAPI (blue). Arrowheads indicate vessels and microvessels **(A–C)** and colocalization **(C, D)**. Scale bars = 20 µm. **(E–G, E′, F′):** MI+EMC heart cross‐sections stained with light green Masson’s trichromic **(E)**, Gallego’s modified trichromic **(F)**, and Movat’s pentachromic stainings **(G)**, and their magnifications **(E′, F′)**. Functional vessels with erythrocytes are observed in the fibrin construct and connecting the myocardium with the fibrin gel **(E′, F′)**. Arrowheads indicate the presence of erythrocytes inside the vessels. Scale bars = 20 µm. **(H):** Histogram showing the vessel density expressed as the percentage ratio between border and remote areas occupied by isolectin‐positive cells in control (MI and MI+Fibrin) and cardiac ATDPC‐treated (MI+Con and MI+EMC) groups. Sham‐operated animals are also present and considered standard references. **(I):** Scar thickness (millimeters) for all infarcted groups. Values are mean ± SEM. ∗, *p* < .05; ∗∗∗, *p* < .0001. All animals were included in the analysis (*n* = 39). Abbreviations: Con, control; cTnI, cardiac Troponin I; DAPI, 4′,6‐diamidino‐2‐phenylindole; EMC, electromechanically conditioned; MI, myocardial infarction alone; MI+Con, myocardial infarction with implantation of fibrin loaded with naïve control cardiac adipose tissue‐derived progenitor cells; MI+EMC, myocardial infarction with implantation of fibrin loaded with electromechanically conditioned adipose tissue‐derived progenitor cells; MI+Fibrin, myocardial infarction with cell‐free fibrin implants; Sham+Con, sham groups that lacked myocardial infarction and underwent implantation of control fibrin‐cell patches; Sham+EMC, sham groups that lacked myocardial infarction and underwent implantation of electromechanically conditioned fibrin‐cell patches.

Fluorescence microscopy demonstrated neovascularization of the fibrin patches in all groups (Fig. 5A–5G; supplemental online Fig. 1). Remarkably, colocalization of the GSLI B4 isolectin endothelial marker and PKH26 within the fibrin construct and the underlying scar suggests that EMC cardiac ATDPCs were integrated into vascular structures (Fig. 5C, 5D). Moreover, we also observed vessel connections between the murine myocardium and the engineered fibrin construct (Fig. 5C, 5G). Refined histological analysis confirmed the presence of erythrocytes inside the vessels within the fibrin construct (Fig. 5E, 5E′, 5F, 5F′), demonstrating the functionality and interconnectivity of these construct neovessels with host tissue circulation (Fig. 5G). SMA was also abundant around scar vessels and engineered fibrin construct neovessels (Fig. 4I–4K; supplemental online Fig. 2).

Vessel density was measured in the border zone of the infarcted tissue. Isolectin staining showed ∼14% greater vessel density in the subjacent myocardium that received fibrin patches loaded with both control and EMC cardiac ATDPCs than from MI controls (*p* = .007 and *p* = .030 for MI+Con and MI+EMC versus MI, respectively) (Fig. 5H). A trend of increase in infarct border zone neovascularization was also observed in the MI+Fibrin group, suggesting that fibrin alone may have angiogenic potential in the ischemic myocardium, an effect that was significantly enhanced when the construct was embedded with cardiac ATDPCs.

Scar volume was measured for each animal by using Masson’s trichrome staining. A moderate decrease in scar size was observed for MI+EMC animals (20.85%) compared with MI animals (26.25%) (supplemental online Fig. 3), not reaching statistical significance.

Scar thickness was also measured for each animal by using Masson’s trichrome staining (Fig. 5I). A relevant increase in scar thickness was observed for MI+Con (0.20 ± 0.04 mm) and MI+EMC (0.23 ± 0.08 mm) animals compared with MI (0.14 ± 0.02 mm) and MI+Fibrin (0.15 ± 0.01 mm) groups (*p* = .001 for MI+Con and *p* < .0001 for MI+EMC versus MI group).

### Implantation of a Physiologically Conditioned Engineered 3D Patch Prevents Ventricular Remodeling and Drives Cardiac Function Recovery After MI

**Figure 6:**
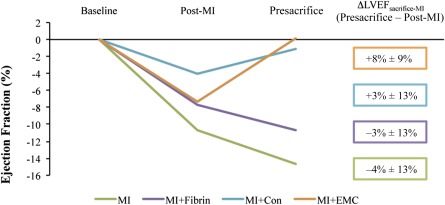
Functional analysis. Left ventricle ejection fraction (LVEF) assessed by echocardiography at baseline, 2 days post‐MI, and at 21 days (presacrifice), in the parasternal short‐axis view, relative to their LVEF value at baseline. Mean values ± SEM. All animals were included in the analysis (*n* = 39). Abbreviations: ΔLVEF, left ventricle ejection fraction differentials between presacrifice and post‐MI; Con, control; EMC, electromechanically conditioned; MI, myocardial infarction alone; MI+Con, myocardial infarction with implantation of fibrin loaded with naïve control cardiac adipose tissue‐derived progenitor cells; MI+EMC, myocardial infarction with implantation of fibrin loaded with electromechanically conditioned adipose tissue‐derived progenitor cells; MI+Fibrin, myocardial infarction with cell‐free fibrin implants.

Echocardiographic analyses were conducted to determine whether EMC cardiac ATDPCs exerted a beneficial effect on the restoration of cardiac function after MI (Fig. 6). Statistical analysis confirmed a similar reduction in cardiac function assessed by left ventricle ejection fraction in all infarct groups (*p* = .14). Significant adverse remodeling assessed by increased ventricular diameters (left ventricle end diastolic diameter and left ventricle end systolic diameter) and depressed function (LVEF and left ventricle shortening fraction) was observed in MI and MI+Fibrin groups; no such ventricular remodeling was present in the MI+Con and MI+EMC groups (Table 1; supplemental online Table 1). The difference in left ventricle ejection fraction (ΔLVEF_sacrifice‐baseline_) between the baseline and presacrifice values was calculated (supplemental online Table 2). Remarkably, 80% of MI+EMC animals presented a ΔLVEF_sacrifice‐baseline_ ≥ −5% (∗, *p* = .03), which is considered clinically relevant. In contrast, only 0% (MI), 33% (MI+Fibrin), and 50% (MI+Con) of the animals not treated with EMC cells presented clinically relevant ΔLVEF_sacrifice‐baseline_ (≥−5%). The LVEF trend among the studied groups is shown in Figure 6. Assessment of ΔLVEF_sacrifice‐MI_ (presacrifice − post‐MI) in MI+EMC animals showed mean values 5%, 11%, and 12% higher than those observed for MI+Con, MI+Fibrin, and MI, respectively (Fig. 6).

**Table 1:** Cardiac function parameters

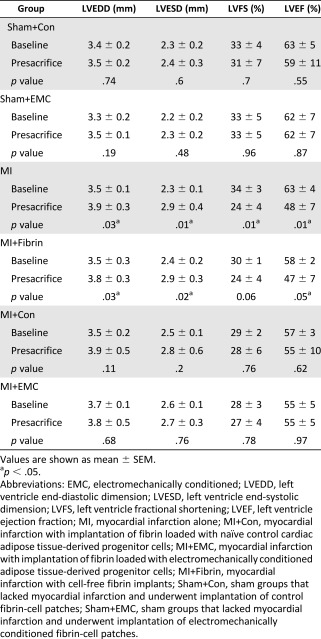

## Discussion

Cardiac cells are normally subjected to electrical and mechanical forces that regulate gene expression and cellular function. The synchronously applied electromechanical conditioning used here was intended to mimic the native cardiac milieu. Both electrical [10] and mechanical protocols were optimized to comply with physiological conditions.

Cardiac and subcutaneous ATDPCs have been described as attractive cell sources for cardiac cell therapy. Indeed, cardiac ATDPCs constitutively express main cardiac genes (GATA‐4, α‐actinin, cTnI, Cx43, and SERCA2), have immunomodulatory properties, and exhibit both cardiomyogenic and endothelial lineage potential when implanted over the infarcted murine myocardium [16, 17, 18]. In a clinical setting, cardiac ATDPCs could be easily obtained through lateral thoracotomy from patients undergoing cardiac surgery. Cells can be expanded and electromechanically conditioned in vitro. Interestingly, the gene expression acquired with the conditioning remained after the stimulation, which would permit cell freezing to preserve them until the conditioned cells are required and delivered during the surgery. Cell delivery could have several approaches, such as the fibrin patch, which is easy to generate once the cells are thawed and is currently being evaluated in a clinical trial [5].

Previously, we explored the effect of electrostimulation alone on cardiac ATDPCs. Relative to EMC cardiac ATDPCs, electrostimulated cardiac ATDPCs exhibited similar expression patterns for the modulation of cardiac transcription factors, but notably lower levels for structural genes [10]. In fact, EMC cardiac ATDPCs exhibit elevated expression of structural and calcium‐handling related genes (α‐actinin, β‐MyHC, and SERCA2), along with decreased expression of MEF2A compared with electrostimulation alone. This comparison suggests that electrical stimulation on cardiac ATDPCs induces early cardiac markers [10], whereas electromechanical stimulation affects both early and late cardiac markers, also detected at the protein level. Moreover, lack of a proliferation marker, such as PH3, and the cTnI expression detected in vivo support a cardiac lineage commitment when EMC cells are embedded in a fibrin patch, which is then implanted in the murine myocardium.

Although individual electrical or mechanical cellular conditioning has been tested previously in vivo [7, 19, 20, 21], this work is the first to combine electrical and mechanical conditioning simultaneously for adipose tissue progenitor cells and use them for therapeutic purposes. Indeed, simultaneous electrical and mechanical stimulation have previously only been analyzed in vitro [13, 15, 22], and this report is the first to study its beneficial effects at the in vivo physiological level. Previous in vitro studies reported improved functional properties and increased SERCA2 and cardiac Troponin T expression after delayed electromechanical stimulation [13]; efficient cardiac differentiation of mesenchymal stem cells when 5‐azacytidine and electromechanical stimulation were applied [22]; and functional maturation of engineered heart muscle with a positive force‐frequency relationship after electromechanical stimulation at 4 Hz [15].

Furthermore, the engineered fibrin construct increased vessel density in the myocardium adjacent to the scar (border region), and this effect was significantly enhanced by the presence of cardiac ATDPCs [17]. Implanted EMC cardiac ATDPCs promoted the sprouting of blood vessels in the underlying myocardium and within the construct. Bayes‐Genis et al. postulated that cardiac ATDPCs might have a paracrine effect, promoting local vascularization by secreting proangiogenic factors under hypoxic conditions [16], and this vasculogenic potential persisted after the electromechanical conditioning. Hoke et al. previously pointed out three putative mechanisms of action for adipose stem cells: myocyte regeneration, neovascularization and paracrine activity. These mechanisms may act cooperatively [23] to prevent ventricular remodeling after MI, an achievement that is crucial to clinical success.

Even though there was no improvement on cardiac ATDPC vasculogenic potential after electromechanical stimulation, their capacity to improve mice cardiac function, compared with nontreated animals, was shown. Mice treated with cell‐enriched engineered fibrin constructs (MI+Con and MI+EMC) exhibited a frank increase in contractile parameters compared with untreated (MI) or cell‐free fibrin constructs (MI+Fibrin) upon sacrifice. However, ventricular remodeling and cardiac function were best with EMC cardiac ATDPC fibrin constructs. The mechanism of action is not fully understood, but as Hoke et al. postulated [23], the cooperation between new functional cardiac‐like cells on the infarction site, increased vascularization on the border region of the scar, and paracrine signaling of implanted cells may help in tissue restoration, and it contributes to the functional improvement. New functional cells, some of them committed to the cardiovascular lineage migrating to the ischemic and healthy murine myocardium, will also benefit myocardial repair. In the same line, neovascularization on scar margins is translated to more oxygen and nutrient supply, which helps in tissue regeneration. Finally, cardiac ATDPC secretion of proangiogenic and cardioprotective factors will enhance cardiac regeneration. In the same line, cell delivery of cells embedded in a fibrin patch was previously reported [17] as a beneficial approach, with slight contribution to cardiac recovery and without harmful effects for the animal.

Only 1 × 10^5^ EMC cardiac ATDPCs induced an 8% improvement of cardiac function. Functional results reported previously [17, 24, 25, 26, 27, 28, 29] used 2‐ to 15‐fold more cells than we used and achieved smaller or comparable improvements. A plausible justification for this enhanced cardiac function in cell‐treated animals is that trained cardiac ATDPCs prevented LV dilation. Indeed, the global functional benefits of the 3D construct may not be restricted to local infarct size reduction, but may also have distant far‐reaching effects on the infarct border zone, as well as on noninfarcted tissue [26, 30]. Together, the significant benefits achieved by this very low cell dose (1 × 10^5^ EMC cardiac ATDPCs) may have a translation to the clinic. It is calculated that humans would need 10^10^ to 10^11^ cells for sufficient engraftment [31], being a prohibitive number for routine cell therapy. Then, the use of EMC cells has the potential to drastically reduce the cell dose, supporting bench to bedside transition.

Host murine cells migrated to the fibrin patch regardless of the presence of embedded cardiac ATDPCs. The migratory cells were stained with antibodies raised against vimentin and SMA to demonstrate the migration of fibroblasts and myofibroblasts to the fibrin constructs. Prior description of the enhancement of fibroblast migration by fibrinogen, especially during healing processes [32], supports this observation.

The ad hoc design of the electromechanical stimulator, which uses 3.5‐cm cell culture plates, limits the number of cells used for implantation, and it is one of our study limitations. A scaled‐up device would allow implantation of a greater number of cells needed for myocardial regeneration for large mammalian hearts, although our physiological conditioning data support beneficial effects with significantly fewer cells. The final time point here was 21 days, comparable with other publications [33]. However, some recommend longer follow‐up times, arguing that midterm data should be interpreted cautiously, yet some differences were no longer significant afterward [34]. Finally, in this study, we did not aim to obtain beating cells, such as neonatal cardiomyocytes or embryonic stem cell‐derived cardiomyocytes. Indeed, we worked with adult progenitor cells, whose cardiovascular potential has already been described [16, 35, 36, 37]. In vivo implantation of EMC cardiac ATDPCs contributes to cell transdifferentiation and electromechanical coupling to the murine myocardium, which may guide the beating frequency and avoid arrhythmias. Adult progenitor cells are easily obtained, and their clinical translation has no ethical concerns. Collectively, our study presents a more committed phenotype of adult progenitor cells toward the cardiovascular lineage, with enhanced potential, and improved cardiac function when implanted on the murine model of MI. Therefore, EMC cells could be suitable for clinical application, yet no ethical concerns apply, and benefits on preclinical models have been shown.

## Conclusion

We report a new protocol for synchronous electromechanical conditioning of adipose tissue‐derived progenitor cells from cardiac adipose tissue, and the use of conditioned cells in an engineered 3D fibrin patch for treating infarcted myocardium in a murine model. In fact, this study is the first to examine the effect of electromechanically conditioned cells in an in vivo scenario. This work provides evidence that electromechanically conditioned ATDPCs maintain their cardiomyogenic potential within the in vivo environment, migrate to the murine myocardium and scar, improve cardiac function after MI, and increase vessel density. Synchronous electromechanical conditioning of ATDPCs before delivery onto infarcted heart emerges as a promising therapeutic strategy to recover cardiac function after MI.

## Author Contributions

A.L.‐V. and C.S.‐B.: conception and design, collection and/or assembly of data, data analysis and interpretation, manuscript writing; C.G.‐M., S.R., C.P.‐V., I.P.‐G., and G.V.‐N.: data analysis and interpretation, manuscript writing; B.S.: data analysis and interpretation; R.B.: conception and design, data analysis and interpretation, manuscript writing; A.B.‐G.: conception and design, financial support, data analysis and interpretation, manuscript writing, final approval of manuscript.

## Disclosure of Potential Conflicts of Interest

The authors indicated no potential conflicts of interest.

## Supporting information

Supporting Information

Supporting Information
