# Supplementary material for: Electromechanical Conditioning of Adult Progenitor Cells Improves Recovery of Cardiac Function After Myocardial Infarction
Source: Stem Cells Transl Med. 2016 Sep 29;6(3):970–81. doi: 10.5966/sctm.2016-0079 (PMC5442794; doi:10.5966/sctm.2016-0079)
Supplement: Supplementary file 1 — Supporting Information [file SCT3-6-0970-s001.pdf]

Supplemental Information - Bayes-Genis et al.

Supplementary Movie. Electro-Mechanical device stimulating ATDPCs.

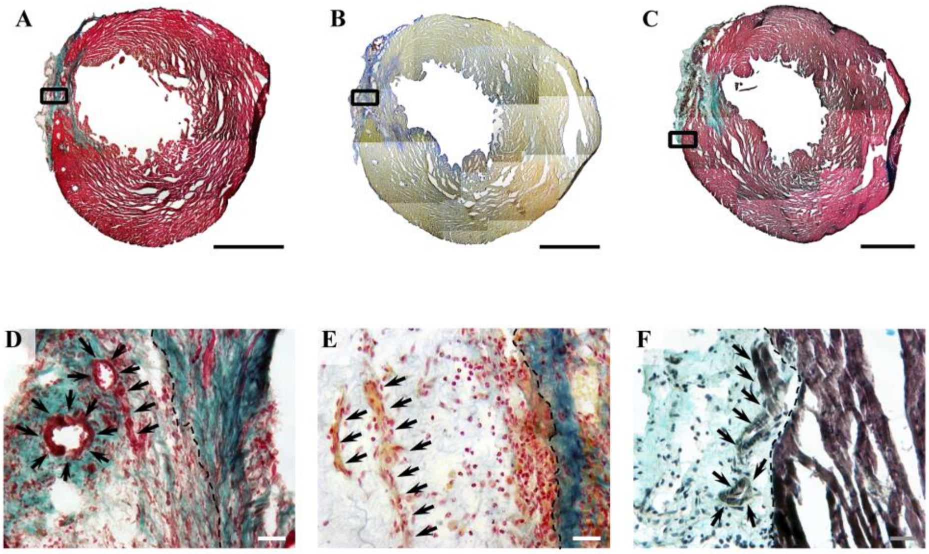

Fig. S1. Histological stainings of MI+EMC heart cross-sections. Light green Masson's trichromic (**A, D**), Gallego's modified trichromic (**B, E**), and Movat's pentachromic (**C, F**) stainings of whole heart cross-sections (upper panels) and magnified fibrin-myocardium interphase showing the vascular structures inside (lower panels). Arrowheads indicate vessels within the fibrin construct. Scale bars = 1 mm (A-C) and 20 µm (D-F).

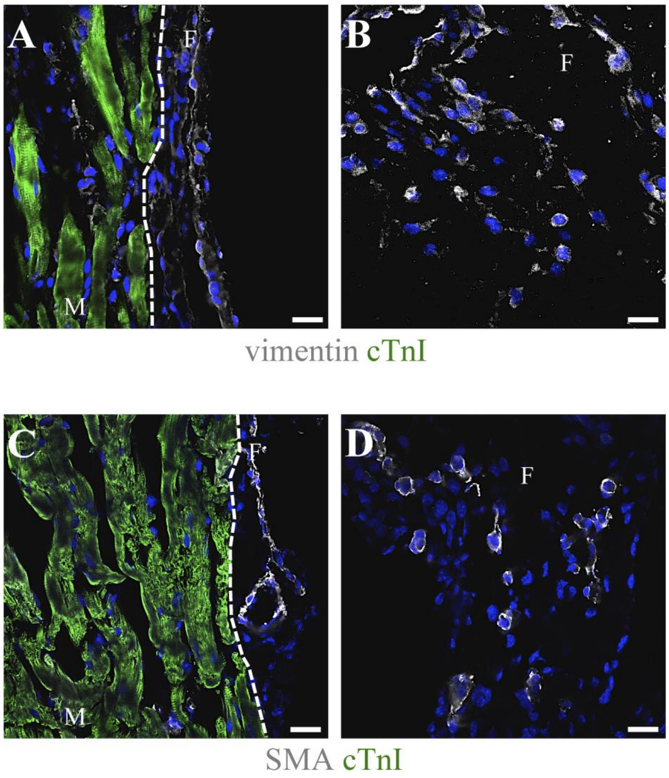

Fig. S2. Immunohistochemistry to assess cell migration from the myocardium to the fibrin patch. Immunofluorescent staining of heart cross-sections in MI+Fibrin animals for cTnI (green), vimentin (grey, upper panels), and SMA (grey, lower panels) in the myocardium-fibrin interphase (**A**, **C**) and inside the fibrin patch (**B**, **D**). Nuclei were counterstained with DAPI (blue). Fibrin = F, Myocardium = M. Scale bars = 20 µm.

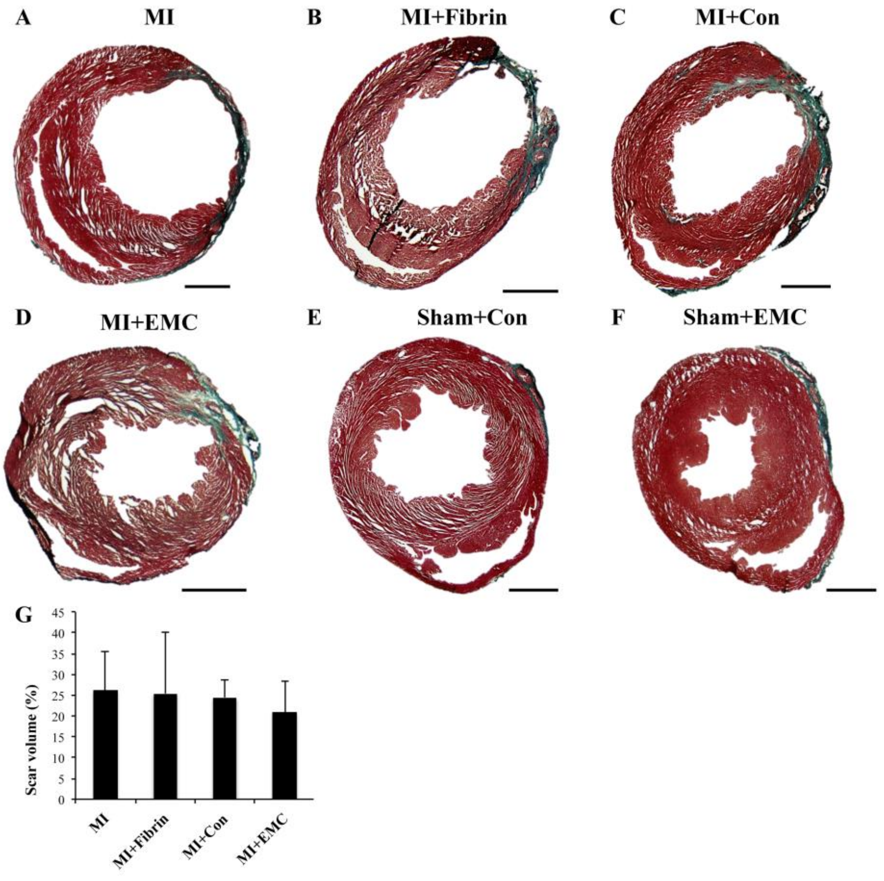

Fig. S3. Morphometric analysis. Representative Masson's trichromic staining of heart cross-section from each animal group: MI (**A**), MI+Fibrin (**B**), MI+Con (**C**), MI+EMC (**D**), Sham+Con (**E**), and Sham+EMC (**F**). Scale bars = 1 mm. (**G**) Scar volume percentages for all infarcted groups. Values are mean ± SEM.

Table S1. Cardiac function parameters.

	Sham+Con			Sham+EMC			MI			MI+Fibrin			MI+Con			MI+EMC		
	**Baseline**	**Pre-Sacrifice**	***P* value**	**Baseline**	**Pre-Sacrifice**	***P* value**	**Baseline**	**Pre-Sacrifice**	***P* value**	**Baseline**	**Pre-Sacrifice**	***P* value**	**Baseline**	**Pre-Sacrifice**	***P* value**	**Baseline**	**Pre-Sacrifice**	***P* value**
LVAWd (mm)	0.9±0.2	0.9±0.1	0.64	0.8±0.1	0.8±0.1	0.68	0.8±0.1	0.8±0.1	0.18	0.9±0.1	0.8±0.1	0.31	0.9±0.	0.8±0.1	0.55	0.7±0.2	0.8±0.1	0.28
LVAWs (mm)	1.0±0.2	1.2±0.2	0.36	1.0±0.1	1.2±0.2	0.46	1.1±0.2	1.1±0.2	0.70	1.2±0.2	1.1±0.2	0.31	1.1±0.2	1.1±0.1	0.52	1.0±0.2	1.1±0.1	0.20
LVPWd (mm)	0.8±0.1	0.8±0.1	0.41	1.0±0.1	1.0±0.1	0.69	0.8±0.1	1.0±0.2	0.09	0.7±0.2	0.9±0.1	0.18	0.7±0.1	0.8±0.3	0.45	0.7±0.1	0.8±0.1	0.10
LVPWs (mm)	1.2±0.2	1.0±0.2	0.06	1.3±0.2	1.3±0.2	0.96	1.1±0.2	1.1±0.2	0.58	1.1±0.2	1.0±0.1	0.77	1.0±0.1	1.1±0.3	0.59	0.9±0.1	1.1±0.1	0.02*

Values are shown as mean ± SEM. LVAWTd: left ventricle anterior wall thickness diastole; LVAWTs: left ventricle anterior wall thickness systole; LVPWTd: left ventricle posterior wall thickness diastole; LVPWTs: left ventricle posterior wall thickness systole.

Table S1. Cardiac function parameters. Values are shown as mean ± SEM. *P < 0.05. LVAWTd, left ventricle anterior wall thickness diastole; LVAWTs left ventricle anterior wall thickness systole; LVPWTd, left ventricle posterior wall thickness diastole; LVPWTs, left ventricle posterior wall thickness systole.

Table S2. Left ventricle ejection fraction calculated as differentials between values at 21 days post-operation and baseline.

	**ΔLVEF (%)**	***P* values**
**MI**	-15±8	
**MI+Fibrin**	-11±8	0.56
**MI+Con**	-3±8	0.23
**MI+EMC**	0±7	0.03*

Values are shown as the differentials in percentage ± SEM. LVEF: left ventricle ejection fraction. *$P < 0.05$; *P* values are referred to MI group.

Table S2. Left ventricle ejection fraction calculated as differentials between values at pre-sacrifice (21 days post-operation) and baseline. Values are shown as the differentials in percentage ± SEM. LVEF, left ventricle ejection fraction. *P < 0.05; P values are referred to MI group.
